# Editorial

**Published:** 1841-11-27

**Authors:** 


THE MEDICAL EXAMINER.

PHILADELPHIA, NOV. 27, 1841.

### OPERATIONS IN COMPLETE ANCHYLOSIS OF THE KNEE.

We had the gratification of witnessing, on Wednesday, Nov. 21st, an operation by Professor Gibson, at the Philadelphia Alms-house Hospital, for the cure of the deformity resulting from perfect anchylosis of the knee-joint in a negro apparently under middle age.

The coalescence of the tibia, patella and femur appeared to be complete, and the leg was fixed in the flexed position, forming an acute angle of about 60 degrees with the os femoris. We arrived, unfortunately, too late to examine the position of the limb with accuracy before the patient entered the anatomical amphitheatre. The operation was of the same character with that first instituted by Dr. John Rhea Barton, and consisted of the removal, by the amputating saw, of a portion of the condyles and body of the os femoris as near as possible to the knee-joint; the fragment removed being angular, with the angle corresponding, as nearly as possible, with the posterior face of the os femoris.

The nicety of the result depends upon the accuracy of the eye of the operator in removing just so much bone as is necessary to bring the limb into an attitude perfectly straight and useful, and upon the close approximation of the incision to the obliterated knee-joint. The latter caution reduces the subsequent deformity resulting from the prominence of the patella—which, of course, remains attached to the lower fragment—to a minimum. It is necessarily considerable, under any circumstances.

The first incision, with the scalpel, commenced on the inner side of the leg, at a point corresponding with a transverse plane touching the posterior surface of the condyles, and was carried directly across the limb, as nearly as possible to the highest anterior elevation of the condyles, to terminate on the out side of the limb, at a point corresponding with the same transverse plane. A second superior incision extended from the inner extremity of the first nearly to its outer extremity, sweeping in an arc across the front of the thigh, so as to include a crescent just sufficient to lay bare the proper amount of bone after the contraction of the skin. These incisions were carried down the bone—the marasmus of the two vasti and the rectus muscles having nearly obliterated them. The flap was then dissected up, from the interior to the exterior angle, and left pendantly the latter, where the crescent was allowed to remain incomplete in order to preserve the vitality of the flap. The bone being thus exposed, the amputating saw was applied, first, nearly in the line of the first incision, and then, as nearly in the direction of the second incision. From below, the saw passed somewhat obliquely upwards, and, from above, with considerable obliquity downwards;—the two planes of division meeting in a line corresponding very nearly with the posterior surface of the bone in the depression between the condyles, and separating a piece including *considerably more than a right angle*, which was rendered necessary by the acuteness of the flexion of the leg. The posterior portions of the scroll formed by the condyles being but partially divided, on account of the proximity of the popliteal vessels—a wound of which would have rendered immediate amputation requisite, of course—these portions were carefully separated through the greater part of their thickness, by means of a narrow-bladed resection saw. The few remaining bony connections between the fragments were then readily broken by increasing, a little, the flexion of the limb.

No attempt was made, immediately, to extend the leg. The flap was returned, over the cavity made by the excision of bone, and its edges were secured, by suture, to the edges of the remaining skin, above and below. The patient was then removed from the amphitheatre, and the limb was placed at rest on a double inclined plane, without any immediate attempt at extension.

It is proper to state that these observations were made at the distance of several feet, and where motives and intentions are given, they are deduced from the language of the operation itself, which was neat, clear and plain, needing no interpreter. This diminishes our regret at the impossibility—from press of occupation—of hearing either the preliminary or the concluding remarks of the operator. This mode of treating anchylosis is novel, having been performed, we believe, but twice upon the knee—once, only, before the present case, by its originator, Dr. Barton. No further apology for the minuteness of the description need be offered. The result will be made known in due time, but we may safely say, even at present, that the proceeding, though apparently formidable, is merciful in conception and trivial in danger to the horrible method of M. Louvier, commented on in number 46, (page 738.) It is applicable, of course, only in complete anchylosis. In that form of the disease in the knee which depends upon muscular contraction and ligamentous changes,—false anchylosis,—the late experience of surgeons in this city, appears to prove that the deformity is always curable, and generally with very little difficulty or pain, by machinery alone: and there is no hardihood in the prediction that the lately favourite plan of cutting the ham strings will soon be classed among the unwarrantable and universally condemned operations.

Several points in this interesting case are worthy of further remark.

The profession is generally aware that, many years ago, Dr. Rhea Barton performed a most ingenious, though severe, operation at the Pennsylvania Hospital, in a case of total loss of motion in the hip-joint, with an unusual position of the limb, which rendered it useless. We have not time, at this moment, to refer to his published description of the case, and memory, at this distance of time, does not enable us to recall the precise cause of the accident; but, the muscles of the thigh and pelvis being in a healthy condition, Dr. B. proposed to divide the os femoris above the lesser trocanter, as nearly as possible to the origin of the capsular ligament, and, by establishing there a pseudarthrosis, to place the limb under the command of the muscles. The operation, though very troublesome and terrible in appearance, was, at first, successful; but, we believe,—standing subject to correction if in error,—that a permanent and inflexible union between the fragments ultimately took place, after an interval of six years, notwithstanding the motions of the limb in walking. The proper direction of the member, however, was effectually restored.

In conversation immediately after the operation, Dr. B. suggested to a few surgical friends the possibility of restoring the usefulness of the forearm by similar means, in cases of complete anchylosis of the elbow-joint. We then expressed our conviction that the plan would not succeed, because the bond of union, under all ordinary circumstances, would either become completely osseous, or, as was more probable, so unyielding from the tenacity of the chondroid or imperfect bone deposited between the fragments, that the muscles, acting, as they must, at great mechanical disadvantage, would prove unable to command the limb. We believe that the experiment has never been tried upon the elbow. When, more recently, Dr. B. first operated upon the knee, the nature of the case did not admit of any hope of the result suggested by his former case. The object was merely the removal of a deformity; and to this extent it is understood to have been beautifully successful. The same remark is true in relation to the operation of Professor Gibson. But whatever may be the present view of the original suggestor of the question, the impossibility of restoring a limb to a certain degree of usefulness by the formation of pseudarthrosis near some of the joints, under favourable circumstances, is not by any means decided. In the knee, however, such a result appears extremely improbable. Independently of the wasting of the muscles—a condition which may be regarded as merely temporary where motion can be re-established—the breadth of the surfaces of bone where the division is necessarily made preclude the possibility of much motion, while their form renders almost impossible the attempt at the formation of a true joint, with a regular capsule, which occurs occasionally—where it is unfortunate instead of beneficial—in pseudarthrosis of the shaft of a long bone.

All that can be reasonably expected—all that *is* expected, we presume,—from the operation immediately under review is, an inflexible and tolerably straight limb. The march of a patient with a perfectly stiff knee is painful and difficult, and the degree in which the comfort of the patient may be really promoted by the operation is a decided, though highly interesting experiment. We shall await, with some impatience, the opportunity of personal observation as to the result, and will then make it the subject of further notice. The actual freedom of locomotion after the recovery, will determine the relative merits of this ingenious mode of treatment, and the amputation of the leg immediately below the knee. For the adoption of the latter method, these cases of complete anchylosis are more favourable than any others, because the obliteration of the joint allows us to remove the leg so completely as to leave no obvious stump, without obliging the patient to bring the pressure of the necessary wooden leg upon parts ill adapted to sustain that pressure. When a patient habitually rests the weight of the body upon the head of the tibia in a kneeling attitude, the skin, instead of ulcerating or becoming tender, is rendered thick and callous; and the construction of wooden legs is now brought to such a perfection that the regular motions of both the knee and the ankle, as well as the combined action of the toes, can be very perfectly imitated at an expense quite within the ability of persons in tolerably comfortable circumstances. We are not perfectly sure that such a wooden leg, which can be used almost without detection, would not be preferable to the natural member with a perfectly stiff knee, for those who can afford from fifty to seventy-five dollars for the purchase of a perfect apparatus.

The chances of life will probably prove to be considerably greater in the operation of Dr. Barton than in amputation, especially if certain cautions adopted by that gentleman and Dr. Gibson should prove to be less important than they are at present esteemed. They will be canvassed presently. The operation *appears* sufficiently formidable, it is true, but, in the wasted condition of the muscles, the parts divided, though bulky, are not of vital importance. No material amount of blood is lost. No great shock is given to the nervous system by the division of important branches. The dangers of secondary hæmorrhage and phlebitis are not enhanced by the injury of large vessels; and the principal danger may be supposed to result from the suppuration of broad surfaces of bone which are not immediately placed in apposition, protracted, *it may happen*, by exfoliation in occasional cases.

The precaution against the immediate or rapid extension of the limb, so decidedly recommended by Professor Gibson, and advocated, if we are rightly informed, by Dr. Barton, is based upon the supposed injury to the popliteal vessels from a sudden change of position. This delay, if necessary, is seriously unfortunate, for it materially protracts the cure, and increases the chance of local and constitutional accidents. We are not prepared to condemn it, but, based, as it is understood to be, upon the accidents to the humeral artery during the forcible reduction of old luxations of the os humeri, two instances of which have been given to the public by the operator, we may safely make a few comments upon it, which will no doubt be estimated at their proper value.

In luxations of the shoulder, the change in the relations of the parts produced by the reduction is very considerable. If the humeral artery should have formed close adhesions to the new formed ligamentous connections of the nascent new joint—which must be torn in the reduction—a portion, and, generally, a small portion, of the artery must suffer either an elongation, *measured by inches*, or a rupture of its coats. The latter is the more probable result. But, in the extension of the leg in the case under review—performed as it is about an axis but little removed from actual contact with the popliteal vessels—the elongation of those vessels, if worthy of calculation at all, *must be measured by lines.*—There is, in this case, less danger of adhesion, because there is no attempt at the formation of a new joint, and there are, usually, no new ligamentous connections distant from the original articulation on pursuing unusual tracks, or placed beyond the cognizance of the surgeon, so as to entangle the vessels. Without pretending, then, to decide that no peculiar formation may render necessary the precaution described in some rare instances—and our examination of the case under review was not sufficiently accurate to warrant a question of its propriety in the present instance—it is legitimate to infer that the speedy—not, perhaps, the immediate—extension of the limb is generally admissible when no very considerable force is required for the purpose. The contraction, or rather the positive shortening of the flexor muscles and tendons, will be found practically to offer sufficient resistance to secure the artery against any danger from too rapid extension; and far be it from us to recommend that this resistance should be overcome *by cutting the ham-strings*, as an ultra tenotomist would be compelled to do for the sake of consistency!

If these views be correct, it will prove advisable in most cases to apply a moderate extending force, almost from the moment of the operation; for the sooner the two surfaces are brought nearly into contact the less will be the extent and duration of the suppuration, as well as the chance of exfoliation and constitutional accidents. The rapidity of cure, and the certainty of firm union—if the latter be really desirable—will be greatly increased by this method of action.

We have extended these remarks much beyond our original intention, but not beyond the merits of the subject. The operation will be repeatedly performed, no doubt, by other surgeons, and happy will it prove if it be always performed with equal skill, and a prospect of as happy a termination. R. C.

---

Through inadvertence, credit was not given to the American Journal of the Medical Sciences, for the article by John B. Zabriskie, M.D., from which extracts were made under the Domestic head in our last number. Such neglects occurring in this Journal are never intentional, and we trust they will be rare.

---

### THE COURTLANDVILLE TRIAL FOR MAL-PRACTICE.

In the number of the Boston Medical and Surgical Journal for the tenth instant, there are some remarks upon this case, which we reviewed in a late number of this Journal (page 712) in a spirit, perhaps, of too great mildness. We were willing to believe, in charity to the profession, that parts of the medical testimony on the trial were incorrectly or incompletely reported; or that the questions were so put by counsel—as very frequently happens—that the general nature of the answers rendered them less directly applicable to the case at issue than they appear to be in the words of the reporter. But the sentiments expressed in the following extract impel us to define *our* position in terms not liable to be misunderstood.

"Prosecutions for mal-practice are pretty much of a piece with those for a breach of promise of marriage, and are looked upon by the discriminating public in a similar light. They are in general a pretext, and that is all, for sponging a little money out of some one who has got more than the plaintiff; although sometimes, were it possible to probe to the bottom of the motive, it would be found to be an arch scheme which for ruining the reputation of the defendant. In all trials for malpractice in medicine and surgery, our sympathies are in the first place enlisted on the side of the defendant, knowing, as we do, from years of critical observation into the history of these litigations, that the public good, humanity, benevolence, philanthropy or any other praiseworthy object, is in most cases entirely out of the question.

We hope this report will have an extensive circulation, as, aside from any local or personal object, it will have the effect of putting surgeons on their guard against unprincipled patients and their special friends."

There can be no doubt of the benevolent motive of our friend the Boston editor, in directing implied censure upon the plaintiff and his "special friends," for we know him for an amiable, kind-hearted, and truly hospitable man. That we have not "shared his board" is owing to no lack of courtesy on his part, and should we ever be compelled to "prove his brand," we trust it will be *in tournament and not in fight*. We can even sympathise with his leaning towards the profession, but cannot forget that the first professional duty is towards the patient; nor have we now to learn that though malignity and sordid calculation are no infrequent instigators of prosecutions for mal-practice, it is quite possible for medical men, in these days of easy graduation and multiplied professorships, to be guilty of culpable neglect, or—in the existing condition of many medical schools—scarcely blameable ignorance. It is not improbable that our friend may be in possession of information in relation to the case which has not reached us, otherwise we are at a loss to account for the implied charges of evil motive in the plaintiff. A latitude was granted in the presence of unprofessional witnesses at one of the consultations, which would not have been permitted by the law of ethics established by Dr. Percival, or tolerated in Philadelphia, though our

"———, Committee men and Trustees"

are not at all remarkable for the absence of curiosity or the presence of delicacy in intermeddling with affairs beyond their knowledge and their province. But there is nothing on the face of the pamphlet of Dr. Shipman that would warrant us in impugning the motives or the conduct of the plaintiff or his friends.

On the contrary—we now feel bound to say that if there exist no errors or confusions in the statements contained in that publication—a position we should be very unwilling to assume in relation to any printed report of an American trial—the practice of Dr. Shipman was, in our opinion, correct in every particular except in those debatable and unessential points which were made the subject of stricture in the article to which reference has been already made. On the other hand, nothing is found in the pamphlet to account for or excuse the condition of the patient as therein represented, from the time when the bone was protruded, after the first reduction, until after the removal of the necrosed portion.

Supposing all the evidence to be correctly and completely stated,—had we been the prosecuting party,—not only should we have avoided requesting the withdrawal of the case, but *we would not have permitted it*,—unless under the dire necessity of a pecuniary disability to proceed,—so long as competent surgical evidence and a tolerably intelligent jury were procurable.

We hope the circulation of the pamphlet, painful *as it must be* to the lovers of professional concord, and, justly or unjustly injurious *as it may be* to the reputation of individuals even among the witnesses, will have the effect of "placing surgeons on their guard," as to the necessity of keeping pace with the advance of science, and being careful of *courts of justice*, whether as principals or deponents. Alas! that we are compelled to speak so plainly!

